# Maternal serum from ‘high-stress’ pregnancies demonstrates bioactivity capable of attenuating neurite growth through a TNFα-dependent pathway in SH-SY5Y cells

**DOI:** 10.1042/BSR20260125

**Published:** 2026-06-18

**Authors:** Clara Deady, Lars Wilmes, Fergus P. McCarthy, Ali S. Khashan, Gerard Clarke, James Keane, Cathal McCarthy, Gerard W. O’Keeffe, Siobhain M. O’Mahony

**Affiliations:** 1 Department of Anatomy and Neuroscience, University College Cork, Cork, Ireland; 2 APC Microbiome Ireland, University College Cork, Cork, Ireland; 3 Department of Psychiatry and Neurobehavioural Science, University College Cork, Cork, Ireland; 4 Department of Obstetrics and Gynaecology, University College Cork, Cork, Ireland; 5 INFANT Research Centre, Cork, Ireland; 6 School of Public Health, University College Cork, Cork, Ireland; 7 Department of Pharmacology and Therapeutics, University College Cork, Cork, Ireland

**Keywords:** inflammation, neurite growth, pregnancy, stress, tumour necrosis factors

## Abstract

Prenatal stress is associated with deleterious neurodevelopmental consequences in affected offspring. Prenatal stress via exposure to high physiological levels of inflammation *in utero* may induce an inflammatory state in the fetal brain. However, inflammation is not only associated with disease-states but also can be seen in a healthy pregnancy. There is limited research examining the potential that exposure to biological mediators of stress may have on neurodevelopment. We aim to determine whether certain circulating biological markers in maternal serum influence neurite growth in partially differentiated SH-SY5Y cells as a potential mechanism impacting neurodevelopment. Blood was collected at 20-weeks’ gestation as part of the SCOPE pregnancy cohort study. These factors, including pro-inflammatory cytokines, markers of tryptophan metabolism, and gut permeability that were previously analysed, were used to stratify women into low (*n* = 10) and high (*n* = 10) biological stress groups. Exposure to the serum categorised as ‘high-stress’ significantly reduced neurite length in comparison to serum categorised as ‘low-stress’, with tumour necrosis factor-α playing a substantial role in mediating this reduction. The ‘high-stress’ serum was subsequently found to increase the levels of phospho-Ser536-p65. Phosphorylation of p65 Ser536 has previously been shown to switch NF-κΒ from promoting neuronal growth, to inhibiting it. The reduction in neurite length seen following exposure to the ‘high-stress’ serum was prevented when NF-κΒ p65 was knocked down. The present study emphasises the potential negative impact that circulating factors may have on neuronal growth, and the mechanism behind it.

## Introduction

*In utero* neurodevelopment is a complex, coordinated, and a time-sensitive process. Prenatal stress can disrupt optimal neurodevelopment, but the exact mechanisms remain unclear [1]. Later, this may present as motor deficits, cognitive impairments, or behavioural difficulties [1–3]. These can occur via disruptions of grey and white matter in key brain regions, negatively influencing connectivity [4]. This has been shown in both preclinical work and in human studies [1,5–9].

Biological stress refers to the physiological response to a perceived threat that disrupts normal homeostasis. The response to this involves the interplay of psychological and physical changes, focused on coping with the perceived threat, responding to the threat, and finally restoring balance within the body. The response to stress is unique between individuals. Our prior work identified a maternal serum stress signature at 20-weeks’ gestation in healthy pregnancies, with elevated tumour necrosis factor-α (TNFα), C-reactive protein (CRP), lipopolysaccharide binding protein (LBP), and soluble CD14 (sCD14) linked to self-reported stress, anxiety, and depression [10].

Previous studies have shown that exposure to elevated levels of inflammation during pregnancy can directly impact the central nervous system, however these inflammatory levels are typically higher than identified in the original Keane et al. study and are often linked to overt disease states [11–13]. This signature may indicate an ‘early warning marker’ of active, biological prenatal stress. Elevated TNFα has been shown to cause tissue damage and apoptosis in cerebral organoids, while concentration-dependent effects inhibit neuronal proliferation, and suppressing TNFα improves neurite complexity in inflammation models [14–17].

Prior exposure to pre-eclampsia serum disrupts neuronal growth by increasing neurite number, branching, and length in E18 cortical neurons and neurite length via interleukin-6 (IL-6) in SH-SY5Y and ReNcell® VM cells, demonstrating maternal inflammatory mediators alter neurodevelopment [18,19]. However, it is not yet known whether maternal serum from pregnancies experiencing high, but non-pathological stress, exhibit similar bioactivity [10]. This represents a critical gap in understanding how maternal stress may shape brain development.

## Hypothesis

We hypothesised that maternal serum, which was derived from biologically ‘high-stress’ pregnancies, may possess bioactive properties that are capable of modulating neuronal morphology. Specifically, we anticipated that exposure of neuronally differentiated SH-SY5Y cells to ‘high-stress’ maternal serum would result in reduced neurite length, potentially mediated through a TNFα-dependent pathway. These findings would implicate maternal stress-associated inflammatory factors as direct modulators of neurodevelopmental processes, providing a mechanistic link between prenatal stress and altered offspring brain development.

## Materials and methods

### Participant enrolment and serum collection

The screening for pregnancy endpoints (SCOPE) study was an international multicentre prospective cohort study of nulliparous singleton pregnancies aimed to develop a screening test to predict adverse pregnancy outcomes [20]. The Cork participant cohort of the SCOPE study was recruited between November 2004 and January 2011 (*n* = 1774). Ethical approval for the SCOPE study was obtained from necessary ethics committees (Cork ECM5 (10) 05/02/08)). Ethical approval for the use of the serum collected for this *in vitro* work was granted by Clinical Research Ethics Committee of the Cork Teaching Hospitals (protocol number: APC1004; approval number: APC-D-14). Informed consent was obtained from all women and participants could withdraw at any time. Exclusion criteria included women at risk for pre-eclampsia, having a small for gestational age infant, or preterm birth.

Previously, we selected a subset of the Cork cohort of the SCOPE study (*n* = 209), which we used in the present study. This subset consisted of 105 women with irritable bowel syndrome (IBS) and a randomly selected 104 women without IBS diagnosis [10]. Blood samples were collected at 20-weeks’ gestation, and serum was stored at −80°C.

### Stratification of samples

Keane et al. previously measured the levels of circulating tryptophan and its metabolite kynurenine, three measures of gut permeability; intestinal fatty-acid binding protein (iFABP), sCD14, LBP, and three measures of inflammation; CRP, IL-6, TNFα. To identify groups in our human whole population, a two-step cluster analysis was performed on the 20-week samples as described previously [21]. Participants were not included in the cluster analysis if any data for the circulating markers were unavailable, leaving us with a new population of (*n* = 148). To ensure independence of input variables, initial step *Z*-scores were calculated on serum readouts using the following formula:

$$Z=\frac{\left(x_{i}-\bar{x}\right)}{\bar{\sigma }}.$$

In which *x_i_* represents the test score of each individual, while $\bar{x}$ and *σ* represent the mean and standard deviation of the population, respectively. Subsequently, a combined *Z*-score for distinct pathological readouts was calculated by using the following formula:

$$Z\text{Combined}=\frac{\sum_{1}^{i}z_{\text{test}}}{\text{Number of tests}}.$$

This combined *Z*-score was performed on parameters related to gut barrier integrity (LBP, CD14, iFABP), inflammation (CRP, TNFα, IL-6), and the kynurenine/tryptophan ratio. IBS diagnosis was not considered for producing the *Z*-score.

For the two-step cluster analysis, the individual values of *Z*_Gut-barrier integrity_, *Z*_Inflammation_, and the kynurenine/tryptophan ratio were used as continuous variables for each individual. For the identification of clusters, Log-likelihood was used as a distance measure for the pre-clustering step, while the Akaike information criterion was used as a cluster criterion to estimate the most appropriate number of clusters.

Cluster 1 was identified as a group with low inflammation, a low kynurenine/tryptophan ratio, and high gut barrier integrity (60.1%). Cluster 3 was identified as high inflammation, high kynurenine/tryptophan ratio, and low gut barrier integrity (11.4%). Cluster 2 was identified as the middle group, with a mix of low and high values (28.3%). ‘Low-stress’ and ‘high-stress’ samples were selected based upon having *Z*-scores aligning with Cluster 1 or 3. The lowest overall samples were selected from Cluster 1 and deemed as ‘low-stress’ (*n* = 10), and the highest overall samples from Cluster 3 were defined as ‘high-stress’ (*n* = 10). The women were clustered based on biological stress alone, as the experience to psychological stress is unique to the individual. Following stratification of the women for our study, psychological stress was also evaluated and there was no correlation between the two. This ensures that any potential effect seen in the present study is solely caused by biological markers.

### Cell culture and treatments

Human neuroblastoma SH-SY5Y cells (ATCC) were cultured in Dulbecco’s modified Eagle’s medium/Nutrient Mixture F-12 Ham’s medium (Sigma–Aldrich). Media was supplemented with 10% fetal bovine serum, 1% penicillin–streptomycin, and 2 mM L-glutamine (Sigma–Aldrich). Cells were maintained in a T25 culture flask (Sarstedt, 83.3910.002) at 37°C and 5% CO_2_. In all experiments, unless otherwise stated, cells were plated at 12,500 cells/cm^2^ once they reached 70%–80% confluency. Retinoic acid (RA) (Sigma–Aldrich) was added daily to induce partial neuronal differentiation [22]. Plates were treated and stored in the dark to minimise light exposure.

Individual serum samples from the 10 ‘low-stress’ or ‘high-stress’ women were applied separately to the cells (*n* = 1 per woman, no pooling of serum), all done in triplicate. Unless otherwise stated, all treatments were administered 24 h post plating and the experiment ended 72 h after the first treatment. Final concentrations used include: 10 μM RA; 20 ng/mL of human TNFα recombinant protein (Fisher Scientific); 3% (v/v) maternal serum, 1 ng/mL of TNFα functional blocking antibody (Sarstedt), and 10 nmol of silencer small interfering RNA (siRNA) RELA (Thermo Fisher, Assay ID: s11914) [19]. If *n* = 10 for both ‘low-stress’ and ‘high-stress’ is not indicated, smaller sample sizes were selected at random and based on sample availability. All experiments however had equal numbers of both ‘low-stress’ and ‘high-stress’.

### Neurite length measurements

To measure neurite length, live-cell imaging was performed using phase contrast on an Olympus IX71 inverted microscope. Five non-overlapping images were taken for each well, and the length of five random neurites was measured per well using ImageJ software. Neurite length was chosen as the main functional readout as previous work has shown that circulating factors in maternal serum alter neurite length [19].

### Blocking TNFα activity

To ensure functionality of the TNFα blocking antibody, 1 ng/mL, 2 ng/mL, and 3 ng/mL were examined against 20 ng/mL of TNFα recombinant protein. Recombinant TNFα was administered 1 h after introduction of antibody. Functionality of the antibody was assessed by measuring its impact on neurite length versus recombinant TNFα treatment and the lowest functional dose was selected for further experiments.

### Knockdown of NF-κB p65

To induce knockdown of nuclear factor kappa-light-chain-enhancer of activated B cells (NF-κΒ) p65, 25'000 cells/cm^2^ cells were transfected 24 h post plating. Transfection complex was made in minimum essential medium eagle (Sigma–Aldrich) and contained 10 nmol siRNA RELA (si_p65) or scrambled siRNA (siSCR) negative control (Thermo Fisher), green fluorescent protein (GFP) using the TransIT-X2^®^ Dynamic Delivery System (Medical Supply Co. Ltd). Transfection complex was left for 30 minutes prior to incubation of cells. Cells were transfected with GFP to visualise transfection efficiency, measure neurite length to confirm no basal changes, and then stained for NF-κB p65 to verify knockdown.

### Cytotoxicity assay

Cellular cytotoxic damage was measured based on extracellular release of lactate dehydrogenase (LDH) using the CyQUANT™ LDH Cytotoxicity Assay Kit (Invitrogen). Cell culture supernatant was collected after the experiment and stored at −20°C. At time of analysis, samples were thawed on ice and 50 μL of the cell media was added to 96-well plate, followed by the addition of 50 μL of the reaction mixture as per the manufacturer’s instructions. Samples were incubated for 30 minutes in the dark at room temperature. To end the reaction, 50 μL of the stop solution was added and the absorbance was measured using the Multiskan FC Microplate Photometer (Thermo Fisher Scientific, 51119000), at an excitation wavelength of 490 nm and an emission wavelength of 680 nm. To determine LDH release, the 680 nm absorbance value was subtracted from the 490 nm absorbance prior to calculation of the % cytotoxicity [19].

### Immunocytochemistry

Cells were fixed in 4% paraformaldehyde for 15 minutes prior to washing in 0.02% triton-X-100 phosphate buffered saline (PBS-T). Non-specific binding was blocked by incubating cells in 5% bovine serum albumin (BSA) for 1 h at room temperature. Cells were then incubated overnight at 4°C in primary antibodies diluted in 1% BSA (primary antibodies used, all 1:500 dilution; β-III tubulin (R&D Systems, MAB1195); NF-κB p65 (Cell Signalling, 6956T); phospho-NF-κB p65 Ser536 (Cell Signalling, 3033S)). Cells were washed in PBS-T and incubated for 2 h in the dark at room temperature with secondary antibody in 1% BSA (secondary antibodies used, all 1:500 dilution; goat anti-mouse Alexa fluor 488 secondary antibody (Invitrogen, A11001); goat anti-mouse Alexa fluor 594 secondary antibody (Invitrogen, A11005); goat anti-rabbit Alexa fluor 594 secondary antibody (Invitrogen, A11012)). Cells were washed once again in PBS-T before a 5 minute incubation with 4′-6-Diamidino-2-phenylindole (DAPI) (1:3000; Sigma) in 10 mM PBS at room temperature in the dark. Cells were washed in PBS and imaged at 20× magnification on Olympus IX71 inverted microscope. Four non-overlapping images were taken per well and mean fluorescence intensity was measured by subtracting intensity of adjacent cell-free regions for four cells per image using ImageJ. During analysis, whole-cell regions identified by DAPI or GFP were included. Data presented as arbitrary units (normalised mean grey values).

### Statistical analysis

Due to small sample size, formal adjustment for potential confounders was not feasible. However, maternal demographic and health characteristics were examined for imbalance between the two groups. The findings are interpreted as exploratory and hypothesis generating. Statistical analysis was performed using GraphPad Prism v8.3.0. Student’s unpaired two-tailed *t*-test or two-way analysis of variance (ANOVA) was performed when required and further analysed using Fisher’s LSD post-hoc test or Sidak’s multiple comparisons test when appropriate. All analyses were performed blind to treatment group. Results deemed significant when *P* <0.05. All data presented as mean ± SEM.

## Results

### Maternal characteristics for all participants in ‘low-stress’ and ‘high-stress’ groups

Clinical, socioeconomic, and demographic information were recorded from each participant. There were no differences between the maternal age, weight, number of cigarettes smoked, or units of alcohol consumed between the ‘low-stress’ and the ‘high-stress’ group. For ethnicity, marital status, years in education, employment status, and socioeconomic index, descriptive data were collected. For these variables, data are displayed as a percentage of that group (Table 1).

**Table 1:** Demographic information for the ‘low-stress’ and ‘high-stress’ groups

	‘Low-stress’ (*n* = 10)	‘High-stress’ (*n* = 10)
Ethnicity	Caucasian (100%)	Caucasian (100%)
Age (Years)	28.6 ± 1	29.3 ± 1.9
Weight (Kg)	68.4 ± 4.3	73.8 ± 2.9
Marital status	Married (100%) Single (0%)	Married (80%) Single (20%)
Years in education	<12 (10%) 12–13 (30%) >13 (60%)	<12 (0%) 12–13 (90%) >13 (10%)
Employment status	Full-time (80%) Part-time (20%) Student (0%) Unemployed (0%)	Full-time (80%) Part-time (0%) Student (10%) Unemployed (10%)
Socioeconomic index	<24 (20%) ≥24 (80%)	<24 (30%) ≥24 (70%)
IBS diagnosis	No (40%) Yes (60%)	No (70%) Yes (30%)
Cigarettes smoked (per day the week leading up to visit)	1 ± 1	1.2 ± 0.7
Alcohol exposure (units during first trimester)	6.1 ± 1.6	5.2 ± 1.4

*Unpaired Student’s t-test. Mean ± SEM.*

### Circulating factors in maternal serum differ between the ‘low-stress’ and the ‘high-stress’ groups

An unpaired Student’s *t*-test revealed significant differences in various biological markers of interest between the ‘low-stress’ and the ‘high-stress’ groups. Of the inflammatory markers measured, CRP (*P* <0.0001) was significantly increased in the ‘high-stress’ group, with no differences in IL-6 (*P* = 0.1058) or TNFα (*P* = 0.0894) between groups. Of the markers of gut health and permeability measured, there was no difference in iFABP (*P* = 0.9899) between groups, but both sCD14 (*P* = 0.0014) and LBP (*P* = 0.0489) were significantly altered between the ‘low-stress’ and ‘high-stress’ groups. Finally, the concentration of L-Tryptophan was significantly decreased in the ‘high-stress’ group (*P* = 0.0269). However, this reduction did not lead to any changes in the metabolite L-Kynurenine between the study groups (*P* = 0.2322). However, the L-Kynurenine/L-Tryptophan ratio was significantly increased in the ‘high-stress’ group (*P* <0.0001) (Table 2).

**Table 2:** Biological markers used to profile the women into ‘low-stress’ or **‘**high-stress’ categories

Biological marker	‘Low-stress’ (*n* = 10)	‘High-stress’ (*n* = 10)	*P*-value
CRP (μg/mL)	1.99 ± 0.51	10.37 ± 1.36	**<0.0001**
IL-6 (pg/mL)	0.22 ± 0.05	1.59 ± 0.80	0.1058
TNFα (pg/mL)	0.26 ± 0.13	0.67 ± 0.19	0.0894
iFABP (pg/mL)	5163.41 ± 554.21	5176.52 ± 851.66	0.9899
sCD14 (ng/mL)	736.34 ± 40.32	981.96 ± 51.19	**0.0014**
LBP (μg/mL)	11.57 ± 0.97	19.33 ± 3.55	**0.0489**
L-Tryptophan (pg/mL)	8065.48 ± 778.57	5710.7 ± 622.5	**0.0269**
L-Kynurenine (pg/mL)	184.65 ± 21.72	223.68 ± 22.90	0.2322
L-Kynurenine/Tryptophan ratio	0.02 ± 0.001	0.04 ± 0.002	**<0.0001**

iFABP: intestinal fatty-acid binding protein; sCD14: soluble CD14; LBP: lipopolysaccharide binding protein; CRP: C-reactive protein; IL-6: interleukin-6; TNFα: tumour necrosis factor-α. Unpaired Student’s t-test. Mean ± SEM. *P <0.05, **P <0.01, ****P 0.0001.

### ‘High-stress’ serum reduces neurite length in differentiated SH-SY5Y cells

**Figure 1:**
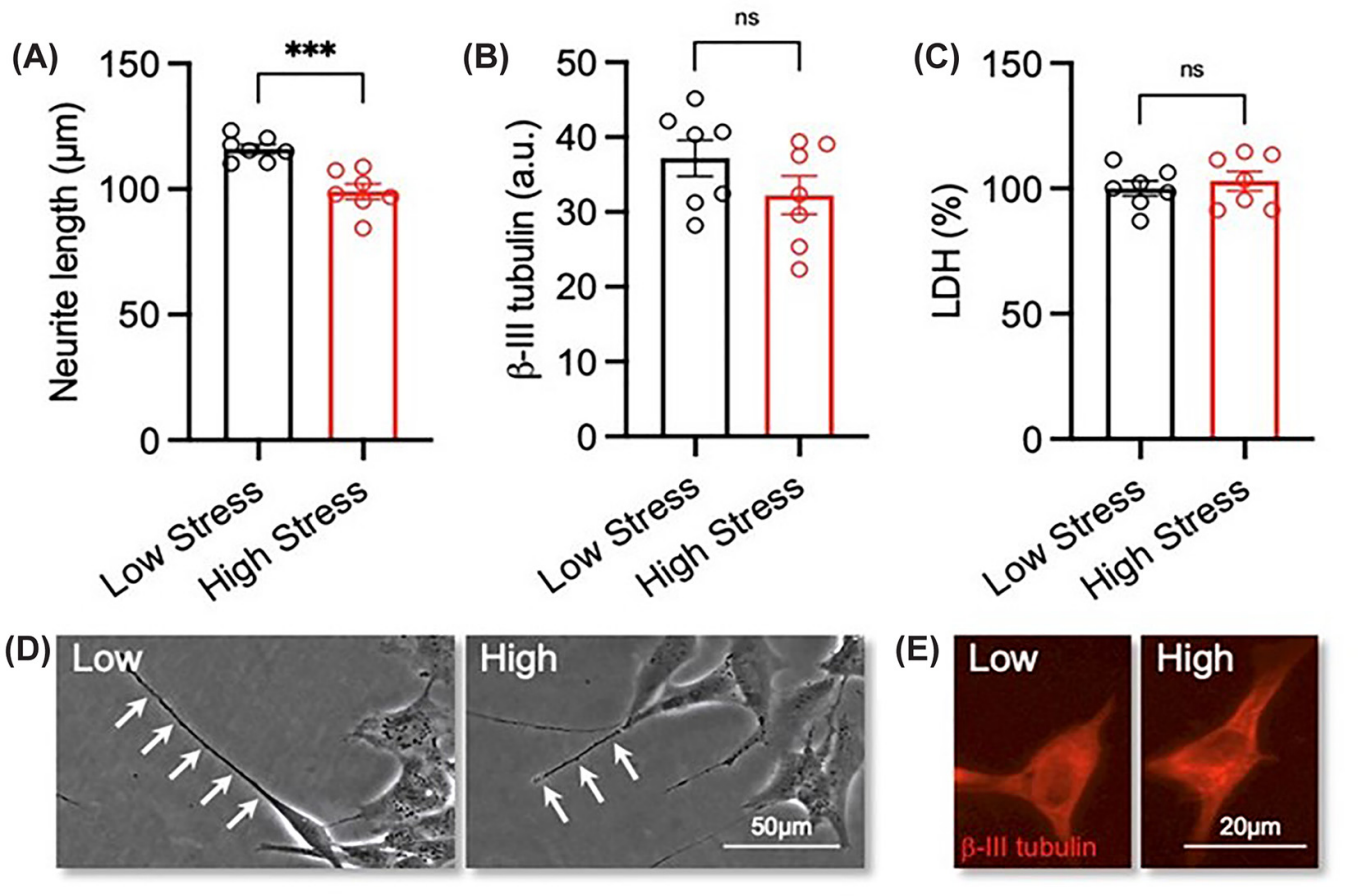
Exposure to maternal serum with a ‘high-stress’ biological signature reduces neurite growth in differentiated SH-SY5Y cells RA-differentiated SH-SY5Y cells were treated with 3% (v/v) serum with a ‘low-stress’ (*n* = 7) and ‘high-stress’ (*n* = 7) biological signature for 72 h. (**A**) Graphs and (**B**) representative phase contrast micrographs of neurite length in each experimental group. Neurites are indicated by white arrow heads. Scale bar = 50 μm. (**C**) Graphs and (**D**) representative photomicrographs of β-III tubulin (red) expression in each experimental group. Scale bar = 20 μm. (**E**) Graph of LDH activity expressed as a percentage of control in each experimental group. Data are presented as mean ± SEM and analysed using Student’s unpaired *t*-test (***P <0.001; n.s. = not significant).

Partially differentiated SH-SY5Y cells were treated with serum from either the ‘low-stress’ and ‘high-stress’ and neurite length was measured post-treatment. Exposure to ‘high-stress’ serum significantly reduced neurite length when compared to the ‘low-stress’ serum (*P* = 0.0005) (Figure 1A,D). This reduction was not associated with changes in β-III tubulin fluorescence intensity, a marker of total tubulin levels (*P* = 0.1880) (Figure 1B,E) or due to a cytotoxic effect of maternal serum as measured using an LDH assay (*P* = 0.5560) (Figure 1C).

### Exposure to ‘high-stress’ serum decreases neurite length in SH-SY5Y cells through a TNFα-dependent mechanism

**Figure 2:**
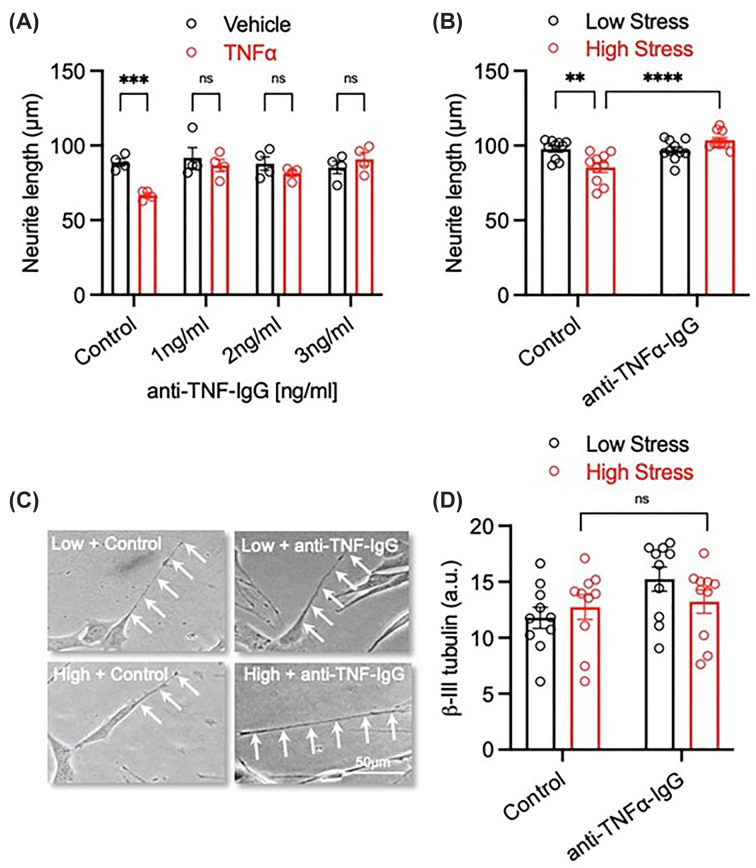
‘High-stress’ serum-induced deficits in neurite growth are mediated via a TNFα-dependent mechanism (**A**) Graph of SH-SY5Y cells treated with 20 ng/mL recombinant TNFα and cultured with or without increasing concentrations (1–3 ng/mL) of a TNFα function blocking antibody (anti-TNFα-IgG) for 72 h. *N* = 4 individual experiments. (**B**) Graph and (**C**) representative photomicrographs of SH-SY5Y cells treated with 3% (v/v) ‘low-stress’ and ‘high-stress’ SCOPE serum and cultured with or with 1 ng/mL anti-TNFα-IgG for 72 h. Scale bar = 50 μm. (**D**) Graph of β-III tubulin expression from the experiment in panel (B). *N* = 10. Data are presented as mean ± SEM and analysed using a two-way ANOVA (**P <0.01; ***P <0.001; n.s. = not significant).

Due to previous work in our lab, we have previously shown that TNFα can have a negative impact on neurite length. Hence, we explored the possibility of TNFα being responsible for the serum-induced reduction in neurite length. To neutralise the activity of TNFα, we examined the efficacy of a functional blocking antibody at various doses in competition with recombinant TNFα protein. There were significant changes in neurite length (interaction (*P* = 0.0228, F(3, 24) = 3.819); antibody effect (*P* = 0.0422, F(3,24) = 3.179); TNFα effect (*P* = 0.0233, F(1, 24) = 5.867)). Sidak’s multiple comparisons test noted a significant difference between the control group and TNFα only (*P* = 0.0035), indicating that the lowest dose of the functional blocking antibody (1 ng/mL) was sufficient to block the effect of TNFα on neurite length (Figure 2A).

Next, we investigated whether blocking TNFα could equally negate the effect of both the ‘low-stress’ and ‘high-stress’ serum on neurite length. A two-way ANOVA revealed significant changes in neurite length (interaction (*P* = 0.0003, F(1, 36) = 16.07); antibody effect (*P* = 0.0006, F(1,36) = 13.96); stress (*P* = 0.2314, F(1, 36) = 1.475)), with Fisher’s LSD post-hoc test noting significant differences between control: low stress versus control: high stress (*P* = 0.0007), control: high stress versus antibody: low stress (*P* = 0.0013), and finally control: high stress versus antibody: high stress (*P* 0.0001) (Figure 2B,C). This result confirms that the higher circulating levels of TNFα in the ‘high-stress’ group plays a role in neurite length reduction. Finally, there were no significant differences in β-III tubulin expression between any groups (interaction (*P* = 0.1631, F(1, 36) = 2.027); antibody (*P* = 0.0665, F(1, 36) = 3.582); stress (*P* = 0.6120, F(1, 36) = 0.2618)) (Figure 2D).

### Serum from ‘high-stress’ serum increased phosphorylation of NF-κΒ p65 Ser536 in differentiated SH-SY5Y cells

**Figure 3:**
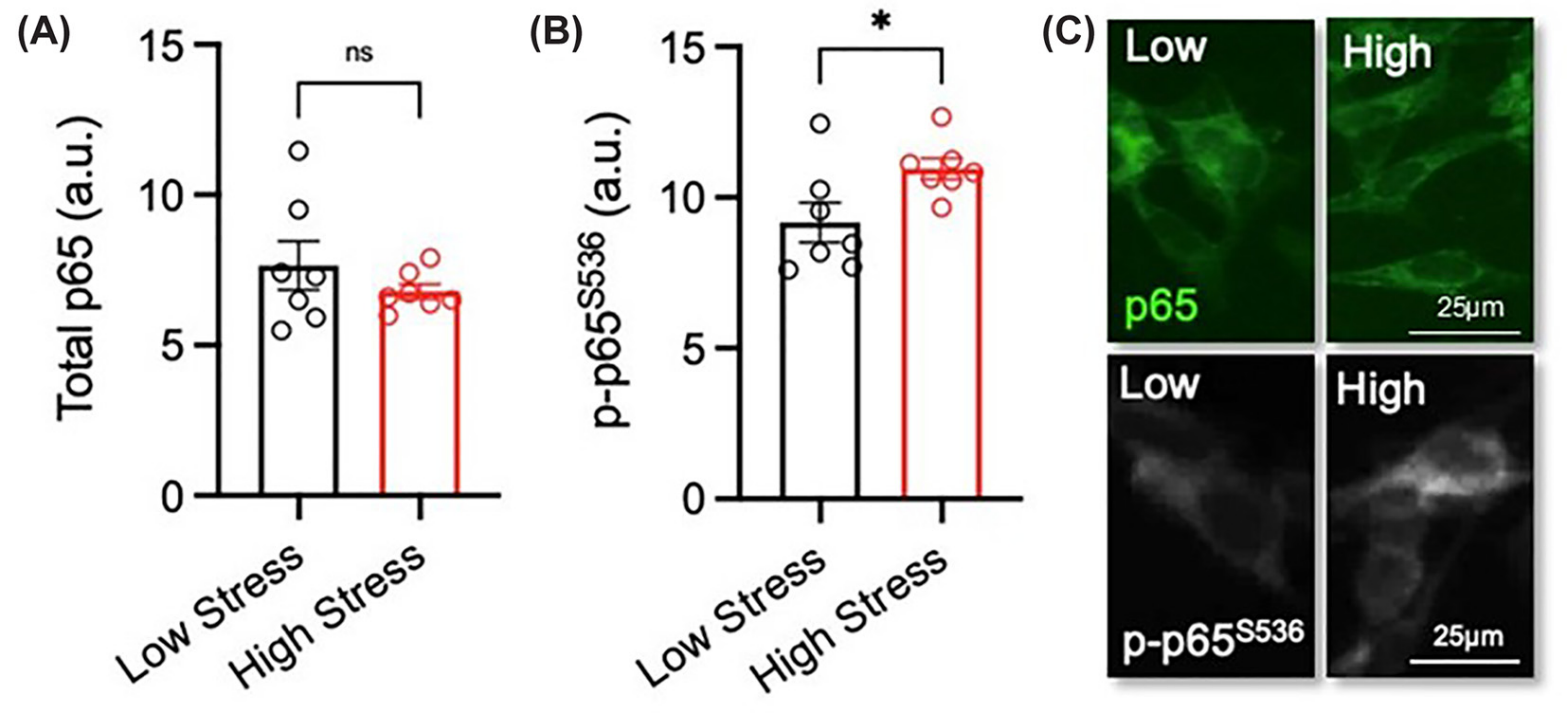
Exposure to serum with a ‘high-stress’ biological signature increases phosphorylation of the Ser536 residue in the NF-κΒ p65 subunit in SH-SY5Y cells RA-differentiated SH-SY5Y cells were treated with 3% (v/v) serum with a ‘low-stress’ (*n* = 7) and ‘high-stress’ (*n* = 7) biological signature for 72 h. (**A–C**) Graphs and representative photomicrographs of (**A–C**) total p65 (green) and (**B,C**) phospho-serine 536 p65 (p-p65S536) (greyscale). Scale bar = 25 μm. Data are presented as mean ± SEM and analysed using Student’s unpaired *t*-test (*P <0.05; n.s. = not significant).

To investigate the mechanism by which TNFα contributes to the reduction in neurite length, we examined possible changes in the status of the NF-κΒ p65 subunit due to its established roles in regulating neural growth [23]. Differentiated SH-SY5Y cells were treated with ‘low-stress’ or ‘high-stress’ serum prior to staining for both NF-κΒ p65 and phospho-NF-κΒ p65 Ser536. While there was no difference in total NF-κΒ p65 expression (*P* = 0.3258) (Figure 3A,C), cells exposed to ‘high-stress’ serum significantly increased phosphorylation of Ser536 residue in the NF-κΒ p65 subunit (*P* = 0.0333) (Figure 3B,C).

### NF-κΒ p65 knockdown prevents reduction in neurite length following exposure to ‘high-stress’ serum

**Figure 4:**
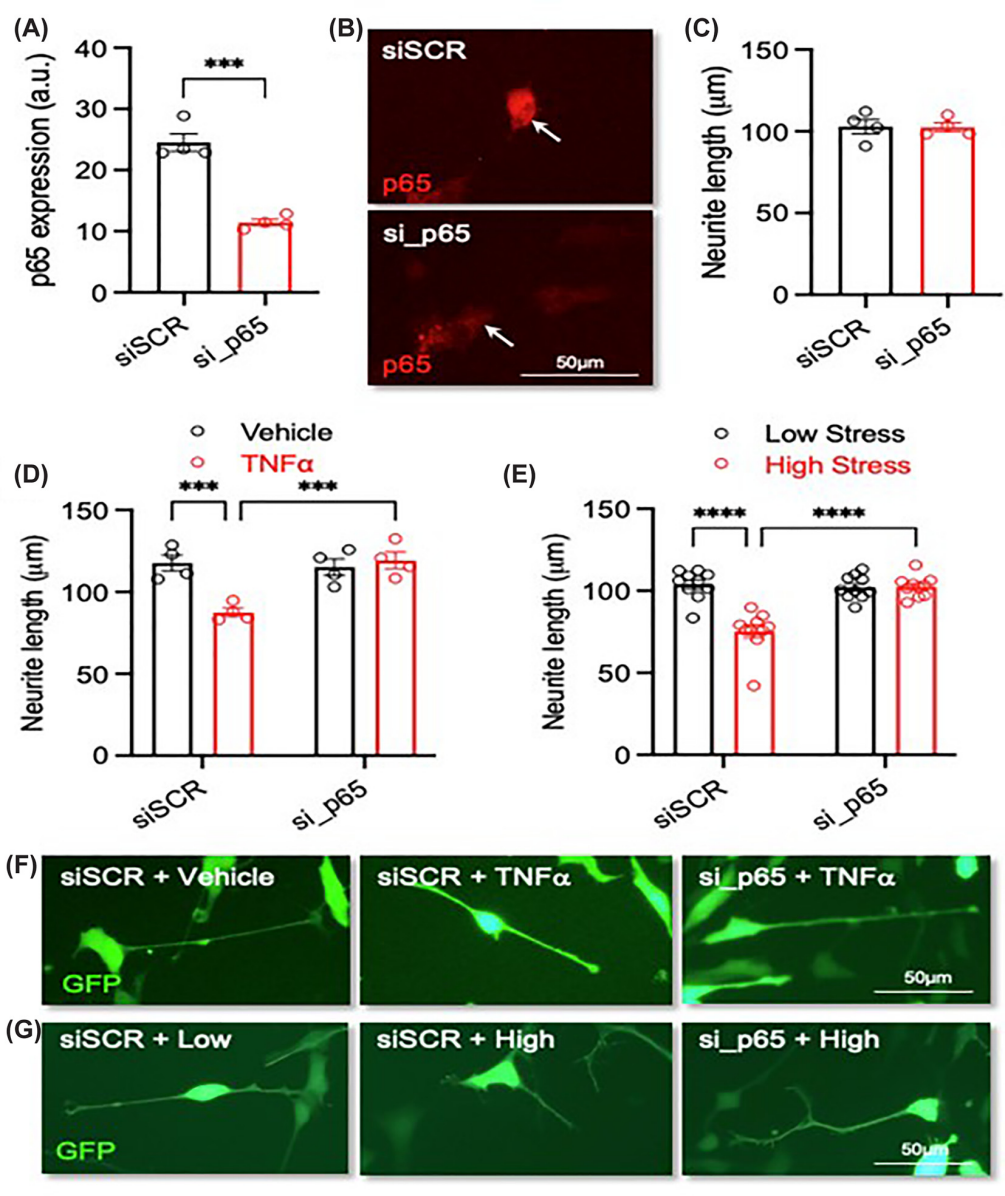
Knockdown of p65 prevents the detrimental effects of exposure to serum with a ‘high-stress’ biological signature on neurite growth (**A**) Graph of p65 expression, (**B**) representative photomicrographs of p65 staining (red), and (**C**) graph of neurite length in RA-differentiated SH-SY5Y cells transfected with 10 nM of a scrambled siRNA or an siRNA against the NF-kB subunit p65 (si_p65) and analysed at 72 h post-transfection. (**D**) Graph of neurite length and (**F**) representative photomicrographs of SH-SY5Y cells transfected with 10 nM of siSCR or si_p65 and cultured with or without 20 ng/mL TNFα for 72 h. *N* = 4 independent experiments. (**E**) Graph of neurite length and (**G**) representative photomicrographs of SH-SY5Y cells transfected with 10 nM of siSCR or si_p65 and cultured in the presence of 3% (v/v) serum with a ‘low-stress’ (*N* = 10) and ‘high-stress’ (*N* = 10) biological signature for 72 h. Scale bar = 50 μm. Data are presented as mean ± SEM and analysed using (A, C) a Student’s unpaired *t*-test or (D, E) a two-way ANOVA with post-hoc Fishers LSD test (***P <0.001; ****P <0.0001; n.s. = not significant).

To further explore the role of NF-κΒ p65 in mediating the serum-induced effects on neurite growth, the NF-κΒ p65 subunit was knocked down via siRNA RELA transfection. Unpaired Student’s *t*-test confirmed successful knockdown via quantification of fluorescence (*P* = 0.0002) (Figure 4A,B). Knock down of the NF-κΒ p65 subunit did not have any impact on basal neurite length (*P* = 0.9413) (Figure 4C). We then investigated whether knockdown of NF-κΒ p65 subunit was sufficient to prevent TNFα-induced neurite length deficits. A two-way ANOVA revealed significant differences between groups (interaction (*P* = 0.0027, F(1, 12) = 14.13); RELA (*P* = 0.0075, F(1, 12) = 10.29); TNFα (*P* = 0.0144, F(1, 12) = 8.16618)) (Figure 4D,F). Fisher’s LSD test showed significant differences between siSCR: no TNFα versus siSCR: TNFα (*P* = 0.0005), siSCR: TNFα versus si_p65: no TNFα (*P* = 0.0011), and finally between siSCR: TNFα versus si_p65: TNFα (*P* = 0.0004) (Figure 4D,F). No significant difference was found between si_p65: no TNFα versus si_p65: TNFα (*P* = 0.5359), indicating that knockdown of NF-κΒ p65 was sufficient to protect against the TNFα-induced defects.

Finally, we investigated whether RELA knockdown of NF-κΒ p65 would protect against the effects of the ‘high-stress’ serum on neurite length. A two-way ANOVA revealed significant changes between the tested groups (interaction (*P* <0.0001, F(1, 36) = 25.03); RELA (*P* <0.0001, F(1, 36) = 19.28); stress (*P* <0.0001, F(1, 36) = 23.64)) (Figure 4E,G). Fisher’s LSD post-hoc noted significant differences between siSCR: low stress versus siSCR: high stress (*P* <0.0001), siSCR: high stress versus si_p65: low stress (*P* <0.0001), and siSCR: high stress versus si_p65: high stress (*P* <0.0001) (Figure 4G). No difference was evident between si_p65: low stress versus si_p65: high stress (*P* = 0.9210) indicating that the reduction in neurite length previously seen following exposure to ‘high-stress’ serum is mediated through the NF-κΒ p65 subunit.

## Discussion

In the present study, we used serum categorised as having a ‘low-stress’ or ‘high-stress’ biological phenotype based on previously defined circulating factors in the maternal blood and assessed their mechanistic role in altering critical neurodevelopmental processes using partially differentiated SH-SY5Y cells.

When compared to the ‘low-stress’ group, exposure to ‘high-stress’ serum significantly decreased neurite growth. This reduction did not appear to be due to cytotoxic damage as confirmed by the secretion of LDH, or disruption to total β-III tubulin expression. To examine the molecular mechanism involved, we used a functional blocking antibody to inhibit activity. Pretreatment of partially differentiated SH-SY5Y cells with the TNFα functional blocking antibody mitigated the effects of ‘high-stress’ serum on neurite growth only.

As TNFα was found to contribute to the negative effect of ‘high-stress’ serum on neuronal growth, we investigated the NF-κΒ signalling cascade, a key mediator of TNFα activity, in particular the p65 or RELA subunit of NF-κΒ. To confirm and elucidate the role of NF-κΒ in mediating the serum-induced effects in neurite length, we used a siRNA approach to knockdown expression of the NF-κΒ p65 subunit. Transfected SH-SY5Y cell cells were then exposed to ‘low-stress’ or ‘high-stress’ serum for 72 h. Knockdown of NF-κΒ p65 subunit rescued the ‘high-stress’ serum induced reduction in neurite length, confirming the specific molecular signalling mechanism involved.

NF-κB is a transcription factor that plays a central role in regulation of numerous genes, involved in immunity and inflammation [24], but its activation has also been shown to promote neurite growth in developing neurons [25]. Yet this can also stunt neurite growth [25]. This paradoxical effect is dependent upon the mechanism of activation involved. Previous work has identified that activation via IκB kinase-β (IKKβ) inhibits neurite growth, and this kinase can be stimulated through TNFα [26]. IKKβ, amongst other kinases, promotes phosphorylation of p65 Ser536 residue, which is crucial to switching NF-κB from a promoting to an inhibitory role in neurons [25]. Furthermore, using S536A, a mutant protein that blocks Ser536 phosphorylation, inhibition of neural growth was abolished [25]. The formation of the phospho–Ser536–p65 complex is key in downregulating genes necessary for promoting growth and upregulating genes that prevent growth [23,25].

The importance of TNFα in having a substantial role in the ‘high-stress’ serum-induced effects on neurite growth is not surprising given its role in systemic inflammation. It is highly plausible that the higher levels of TNFα seen in the ‘high-stress’ serum are influencing other circulating factors in the maternal serum and causing a cascade of downstream inflammatory factors. CRP was also found to be elevated in the ‘high-stress’ serum and may be exacerbating the effects of TNFα. These cytokines have a bidirectional relationship and can sustain the pro-inflammatory environment [27–29]. However, if this is the case, in this experiment TNFα was the primary instigator of the reductions in neurite length as no changes were observed when the activity was blocked.

While the present study’s results allow us to infer the potential role that specific factors may have on the developing brain, it is important to note several study limitations. The present work was performed in SH-SY5Y cells, comprising of both neuroblast-like and epithelial-like cells. Following treatment with RA, neuroblast-like cells are partially differentiated into a more neuronal phenotype, but importantly differentiation consistency was not confirmed, and these cells are not fully neuronal. The use of SH-SY5Y cells in neurodevelopmental studies can be seen as an initial ‘screening tool’ to probe mechanisms and is the first step before progressing to more representative cellular models. Furthermore, our relatively small participant sample size and inability to examine potential confounders acts as a limitation of the present study.

## Future directions

To further understand the effect that ‘high-stress’ serum may have on neurodevelopment and combat these limitations, future studies should incorporate more refined *in vitro* models of the fetal brain, such as embryonic cells, which were not employed in the present study. Primary cells remain an excellent model due to the mix of neurons and glia, enabling thorough examination of complete neurological networks. Future work should examine the effect that TNFα may be having on other inflammatory markers, for instance, does maternal serum increase the number of inflammatory-based cells in the culture or is there a synergistic or additive effect with the other pro-inflammatory cytokines, IL-6 and CRP. Building on this, it would be of interest to examine the developmental mechanistic switch in phospho–Ser536–p65 activity in human stem cells as the previous work was performed in primary neurons isolated from animal models [24]. It would also be interesting to perform a similar experiment for the other circulating factors in the maternal serum, particularly IL-6 and CRP. This would allow us to confirm TNFα as the sole contributor to the cellular changes evident in neurite length or whether it is just a primary instigator. The effect of inflammation on the fetal brain can lead to deleterious outcomes and considering most of these ‘high-stress’ women are classified as healthy with the exception of an IBS diagnosis, it is important to understand the potential risk that elevated levels of pro-inflammatory cytokines may be having in this context.

## Conclusion

In summary, we stratified healthy pregnancies into biological ‘low-stress’ or ‘high-stress’ phenotypes based on defined circulating factors identified in the maternal serum. The biological factors used to stratify the women are all crucial for maintaining optimal fetal health and may play a role in neurodevelopment. When applied to partially differentiated SH-SY5Y cells, the ‘high-stress’ group reduced neurite length, which was confirmed in part due to elevated levels of TNFα. The ‘high-stress’ serum contributed to these deficits through increased phosphorylation of NF-κΒ p65 Ser536, which when knocked down, mitigated neurite length reduction evident upon exposure to maternal serum. These findings offer valuable insights into how exposure to ‘high stress’ during pregnancy can impact neurodevelopment.

## Data Availability

The data from the present study are not publicly available but will be made available upon reasonable request from the corresponding author.

## Abbreviations

ANOVA: Analysis of variance
BSA: Bovine serum albumin
CRP: C-reactive protein
DAPI: 4′-6-Diamidino-2-phenylindole
GFP: Green fluorescent protein
IBS: Irritable bowel syndrome
iFABP: Intestinal fatty-acid binding protein
IKKβ: IκB kinase-β
IL-6: Interleukin-6
LDH: Lactate dehydrogenase
LBP: Lipopolysaccharide binding protein
PBS-T: Triton-X-100 phosphate buffered saline
RA: Retinoic acid
sCD14: Soluble CD14
SCOPE: Screening for Pregnancy Endpoints
siRNA: Small interfering RNA
siSCR: Scrambled siRNA
TNFα: Tumour necrosis factor-α
